# Supplementary material for: Genome skimming and microsatellite analysis reveal contrasting patterns of genetic diversity in a rare sandhill endemic (Erysimum teretifolium, Brassicaceae)
Source: PLoS One. 2020 May 27;15(5):e0227523. doi: 10.1371/journal.pone.0227523 (PMC7252598; doi:10.1371/journal.pone.0227523)

S1 Figure

A.

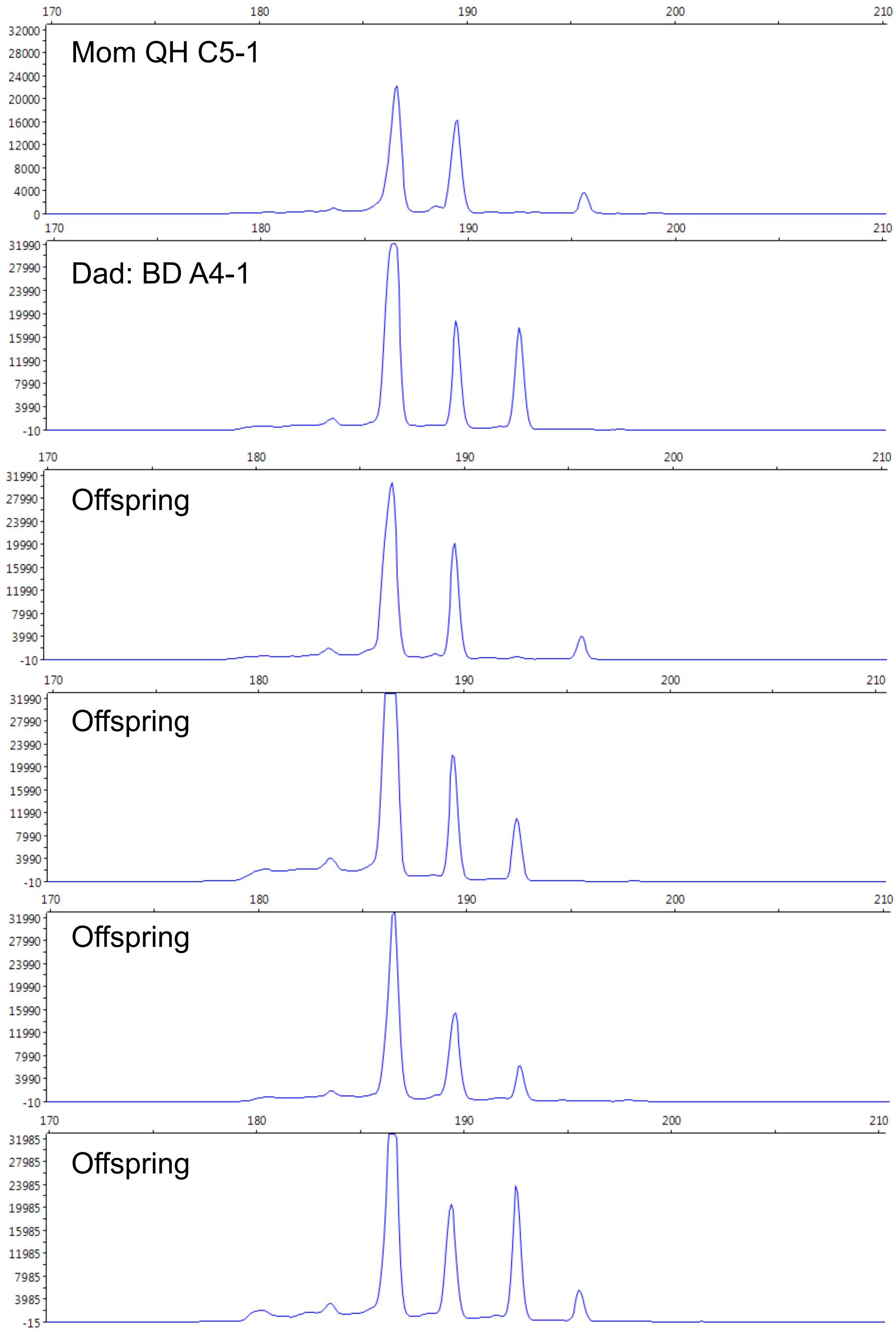

Mom QH C5-1
Dad: BD A4-1
Offspring
Offspring
Offspring
Offspring

S1 Figure

B.

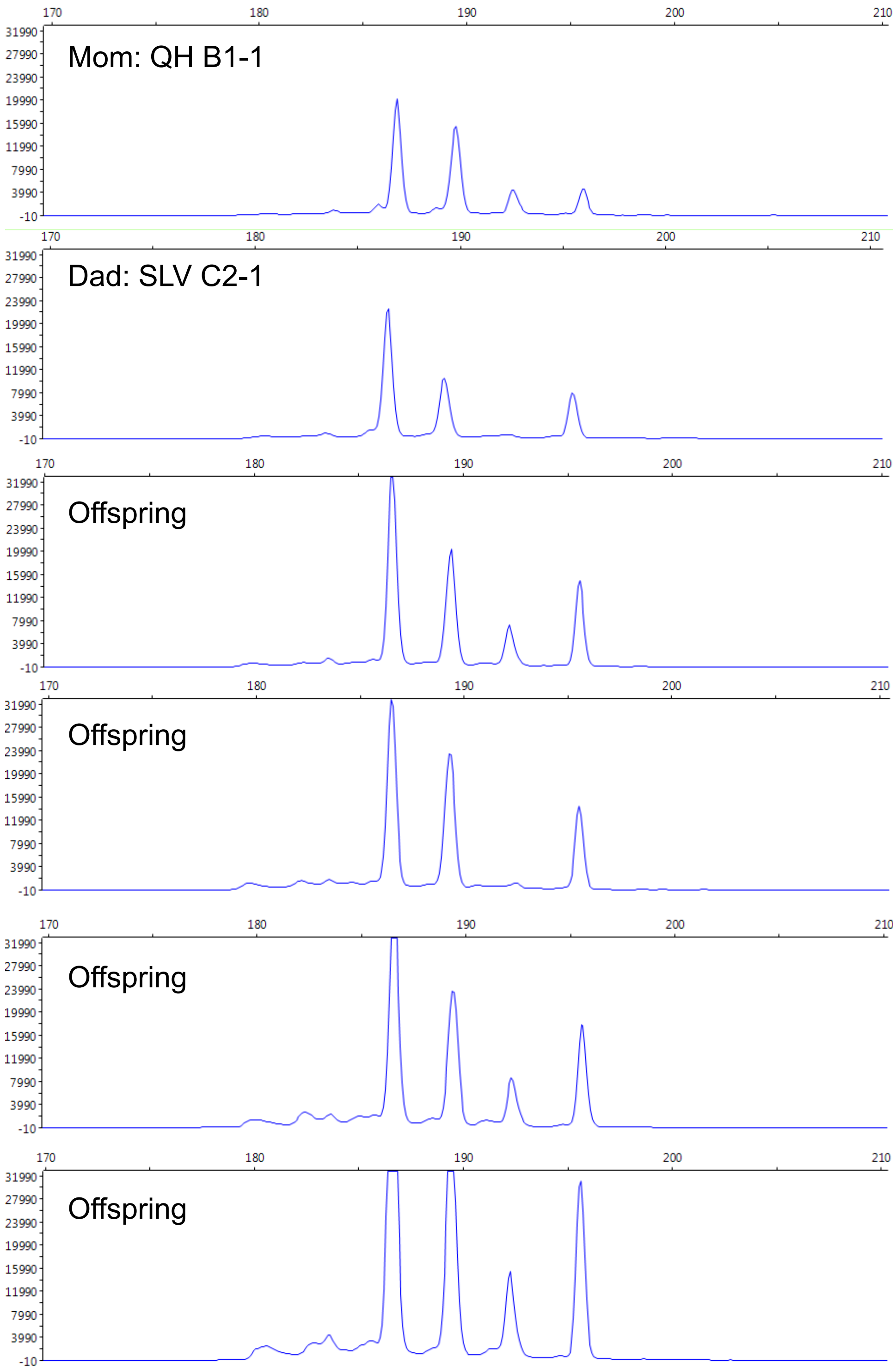

Mom: QH B1-1
Dad: SLV C2-1
Offspring
Offspring
Offspring
Offspring
170
180
190
200
210
31990
27990
23990
19990
15990
11990
7990
3990
-10

S1 Figure

C.

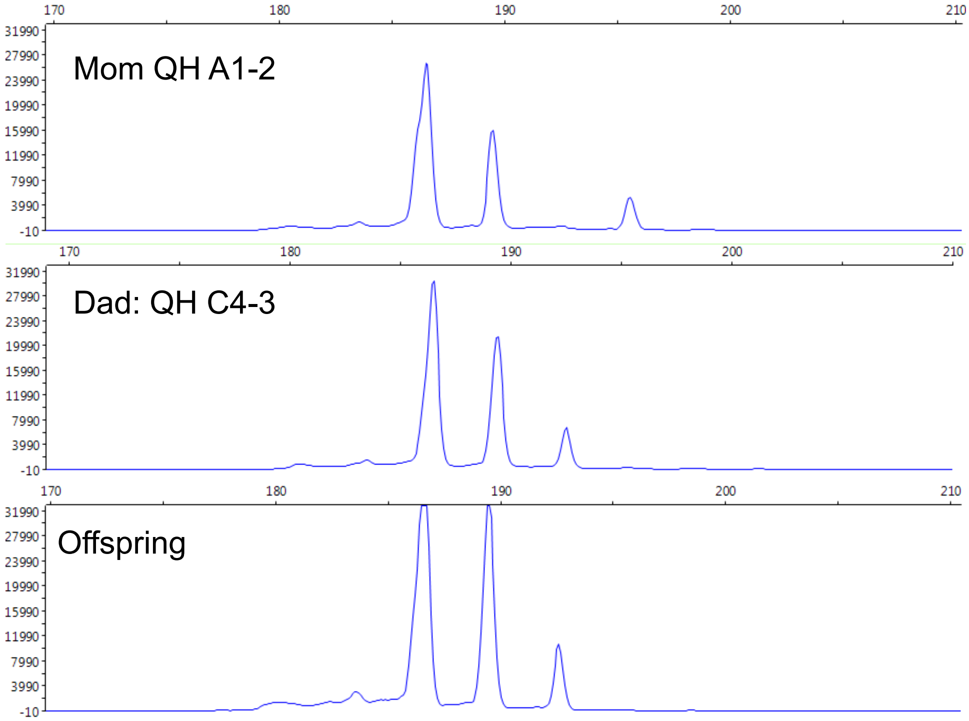

Mom QH A1-2
Dad: QH C4-3
Offspring
170
180
190
200
210
31990
27990
23990
19990
15990
11990
7990
3990
-10

S1 Figure

D.

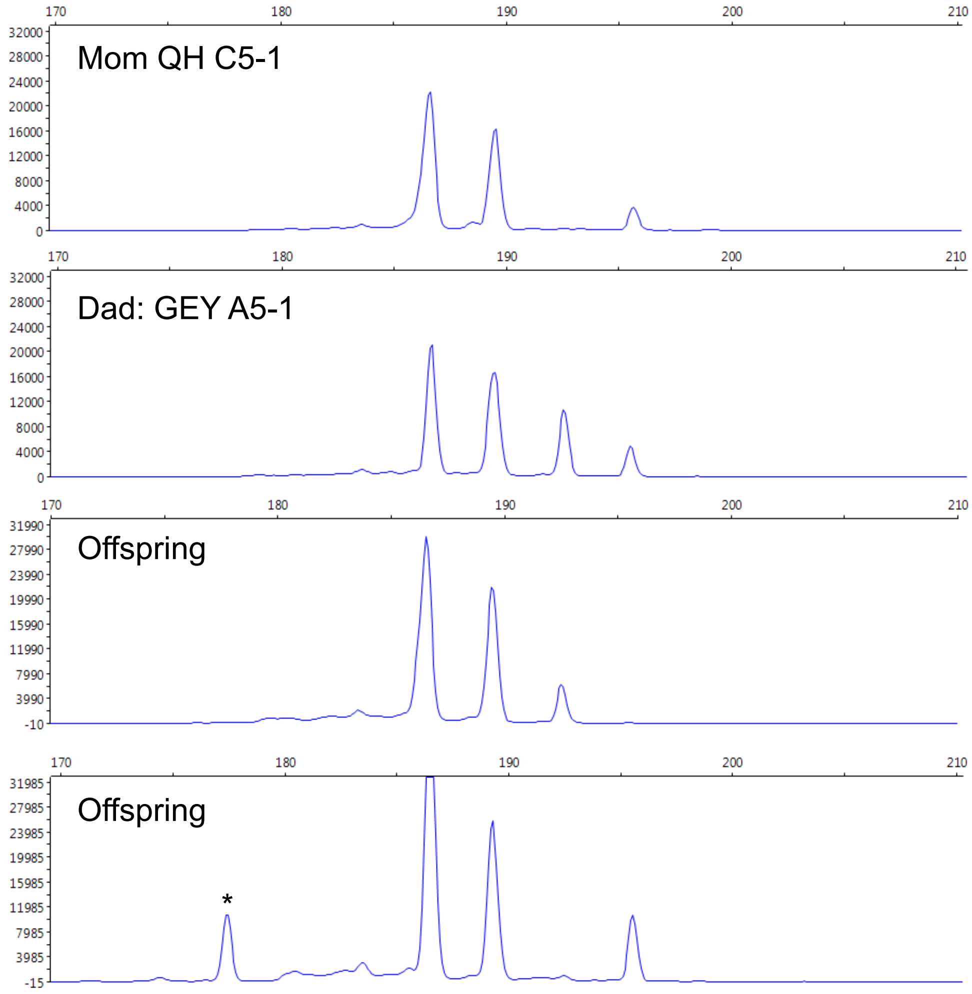
Mom QH C5-1
Dad: GEY A5-1
Offspring
Offspring
*
170
180
190
200
210

S1 Figure

E.

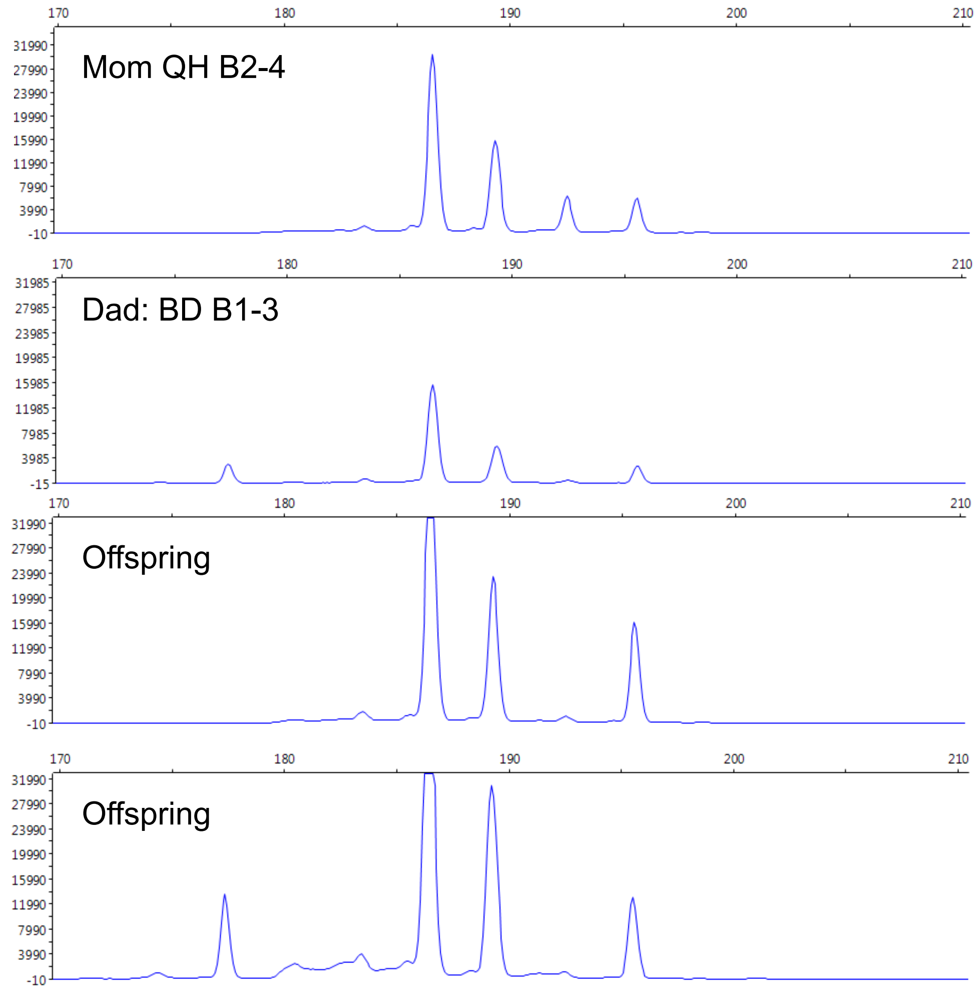

Mom QH B2-4
Dad: BD B1-3
Offspring
Offspring
170
180
190
200
210
31990
27990
23990
19990
15990
11990
7990
3990
-10
31985
27985
23985
19985
15985
11985
7985
3985
-15

S1 Figure

F.

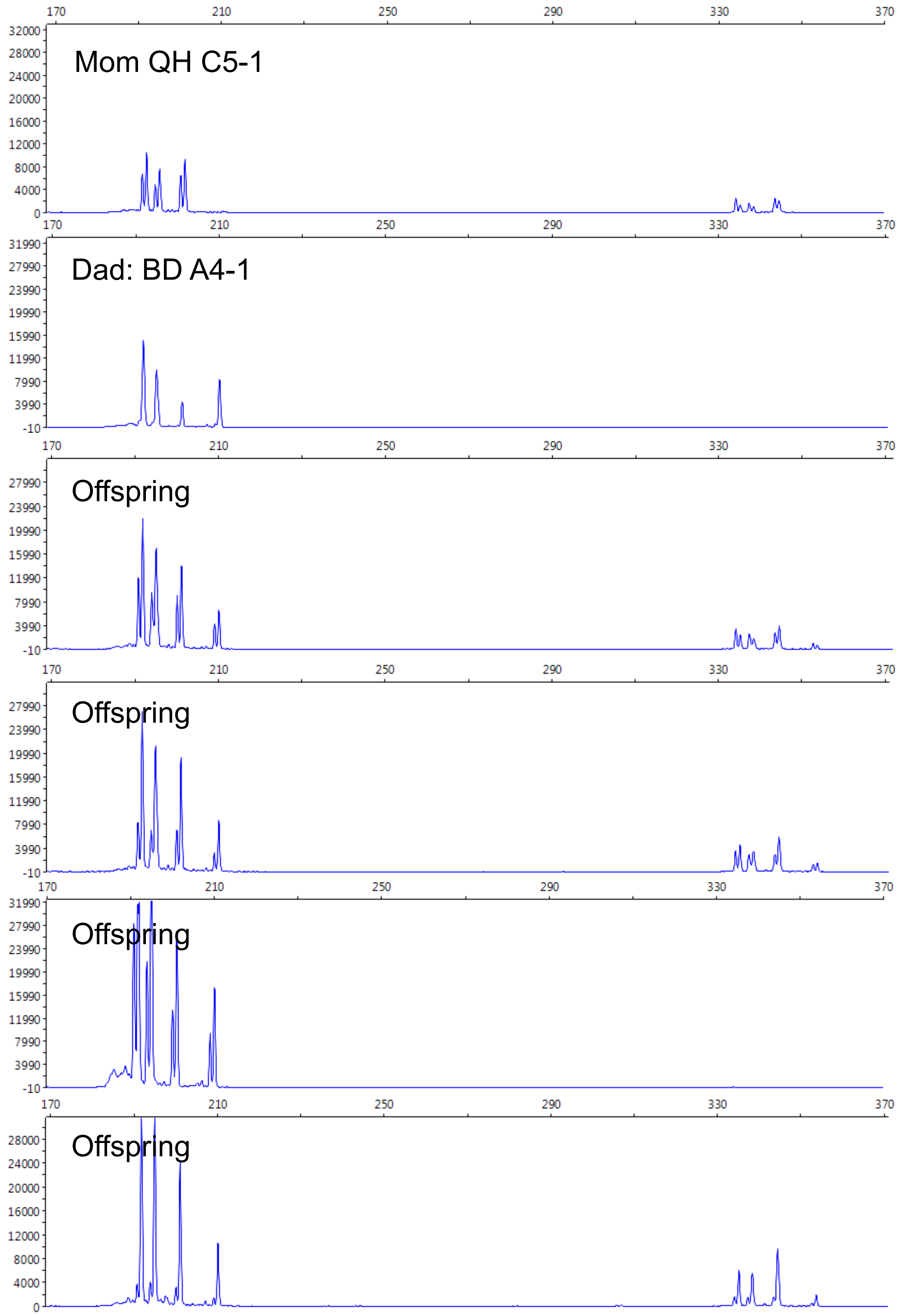
Mom QH C5-1
Dad: BD A4-1
Offspring
Offspring
Offspring
Offspring

S1 Figure

G.

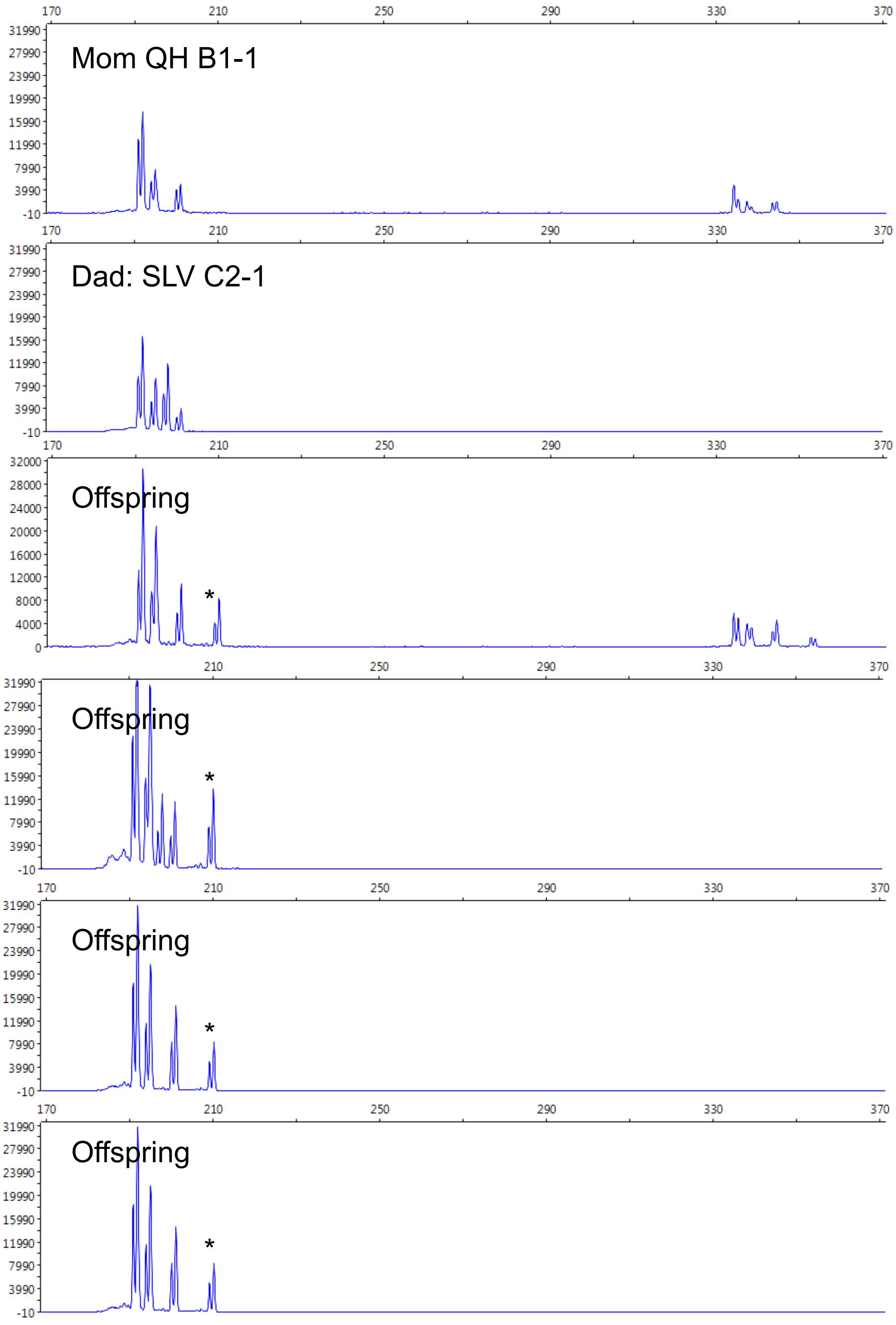
Mom QH B1-1
Dad: SLV C2-1
Offspring
Offspring
Offspring
Offspring
*
170
210
250
290
330
370
31990
27990
23990
19990
15990
11990
7990
3990
-10
32000
28000
24000
20000
16000
12000
8000
4000
0

S1 Figure

H.

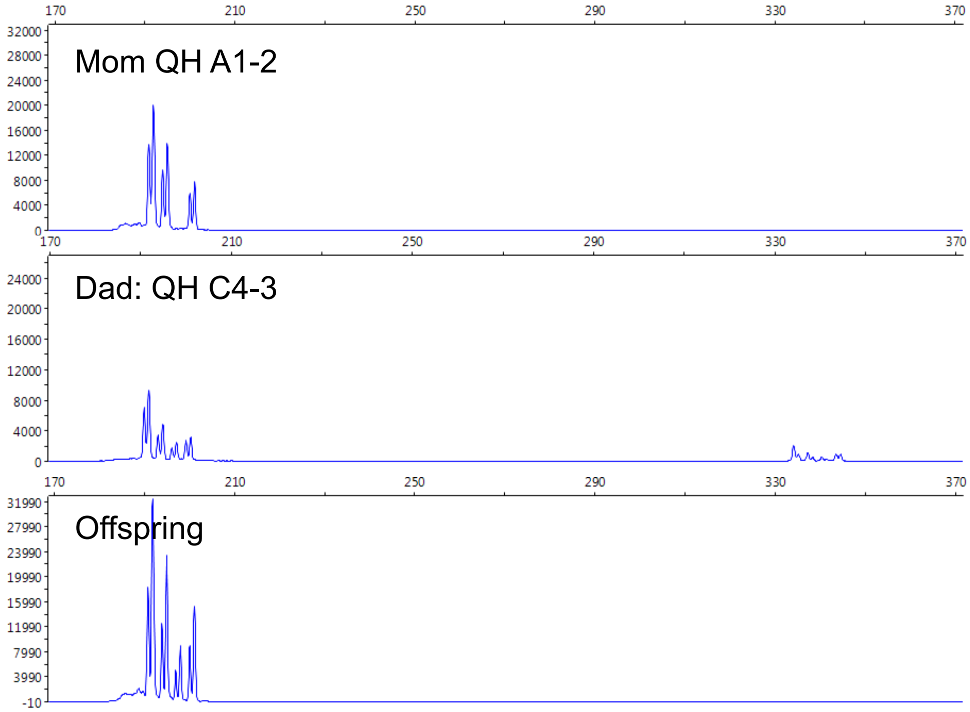

S1 Figure

I.

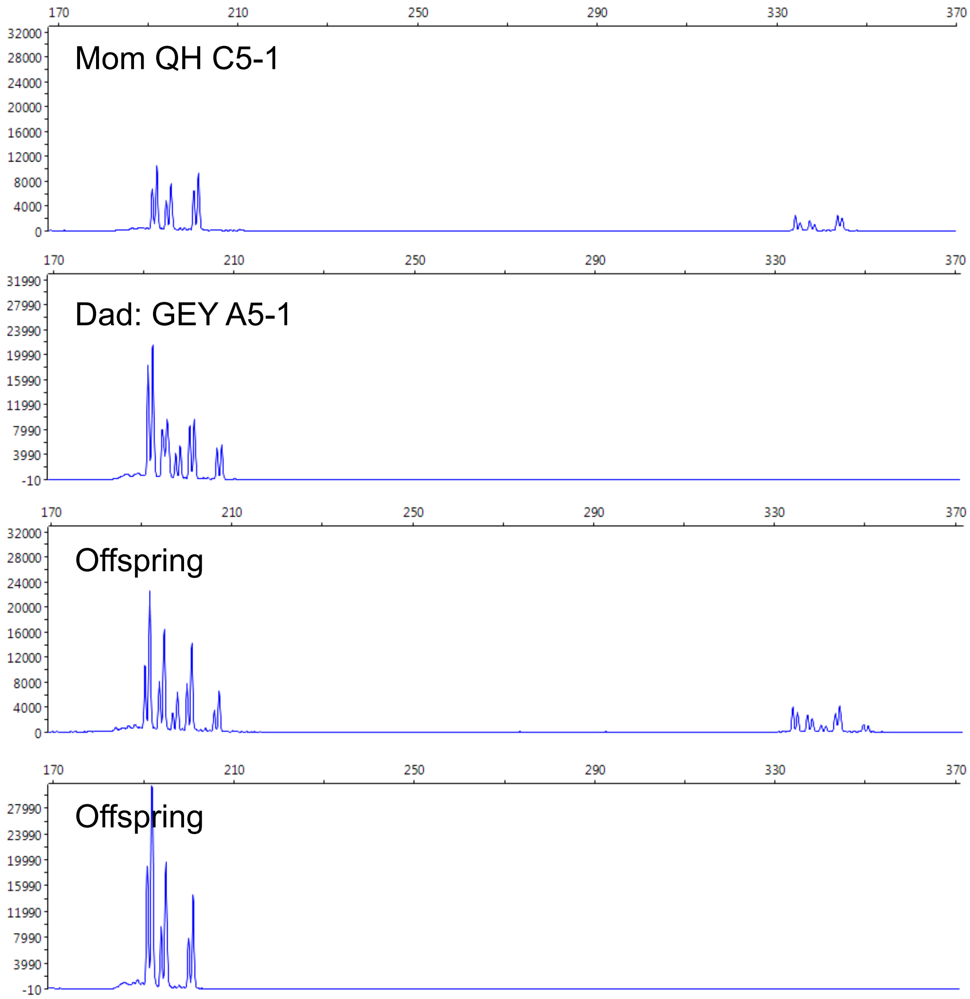
Mom QH C5-1
Dad: GEY A5-1
Offspring
Offspring
170
210
250
290
330
370

S1 Figure

J.

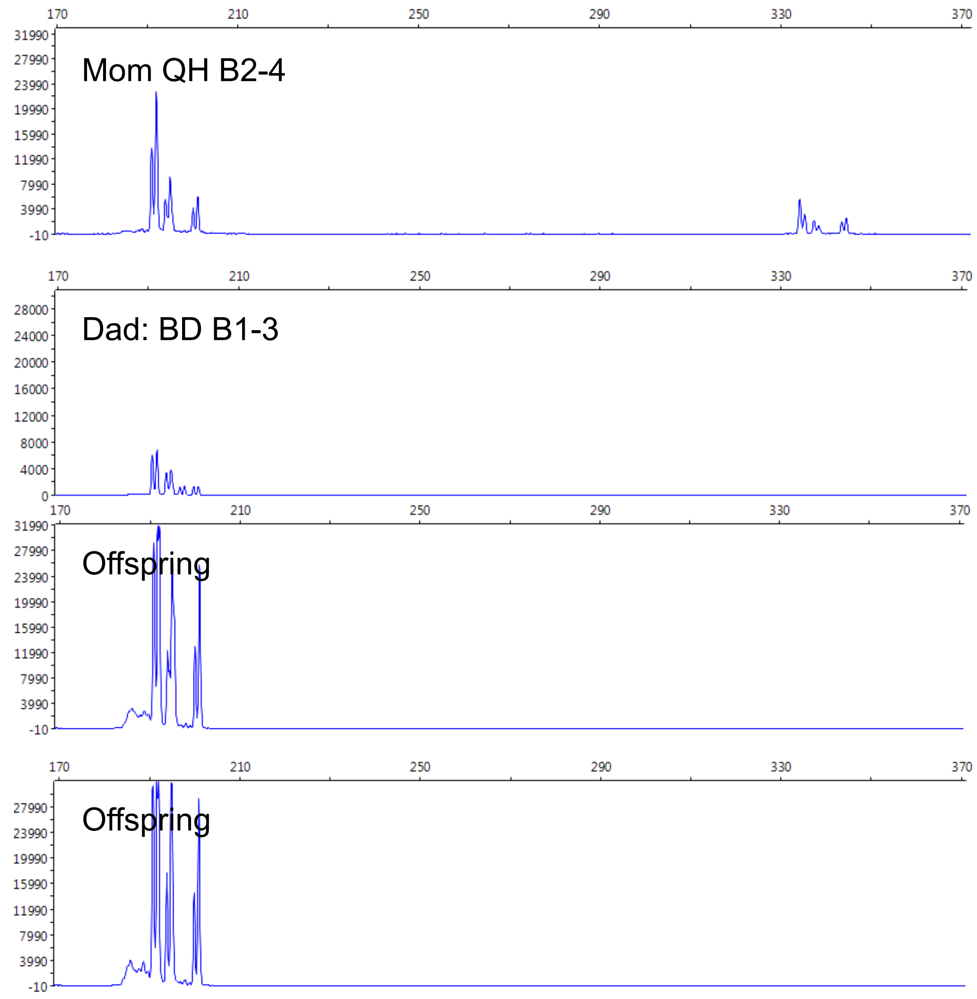

S1 Figure

K.

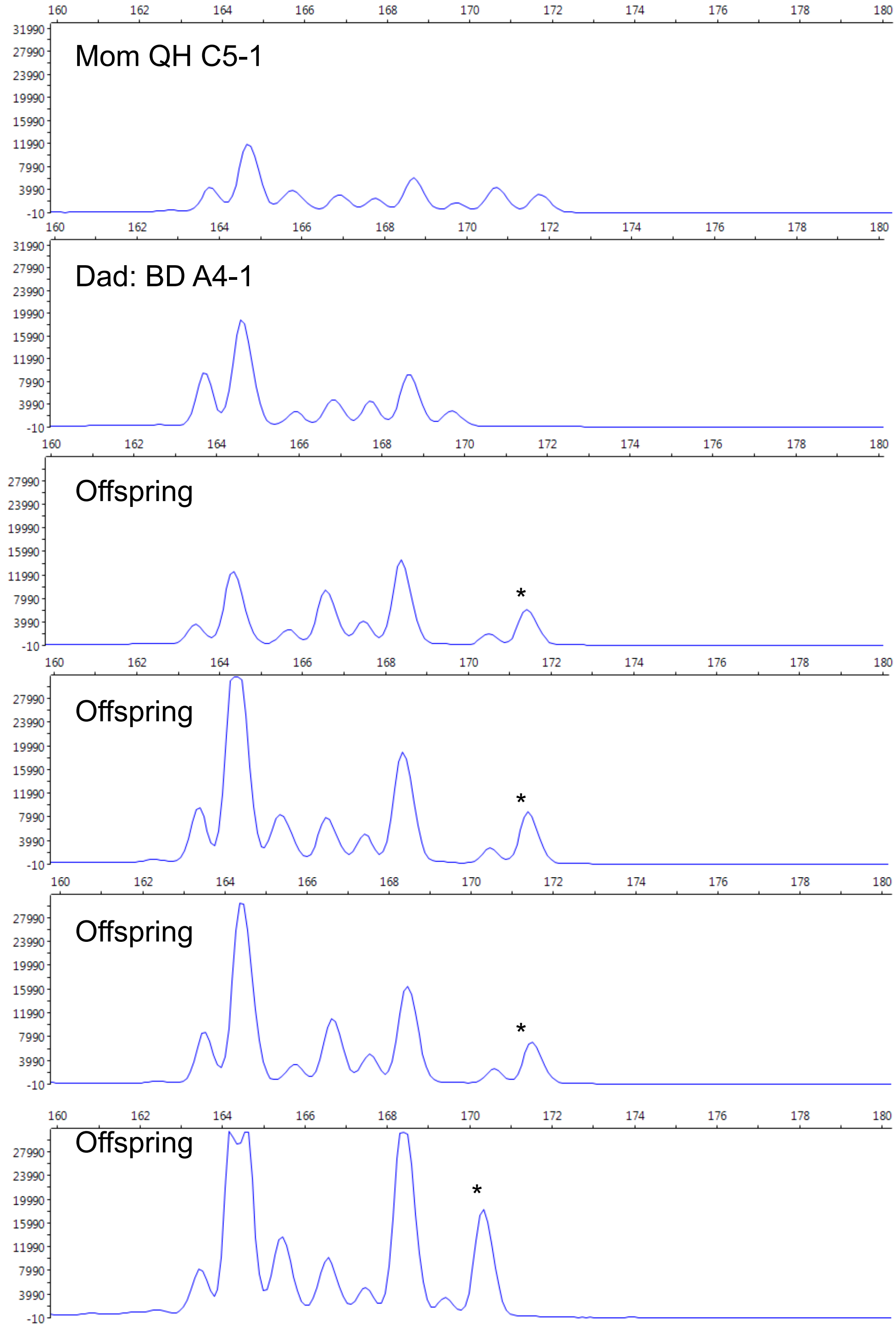
Mom QH C5-1
Dad: BD A4-1
Offspring
Offspring
Offspring
Offspring
160
162
164
166
168
170
172
174
176
178
180
31990
27990
23990
19990
15990
11990
7990
3990
-10
*

S1 Figure

L.

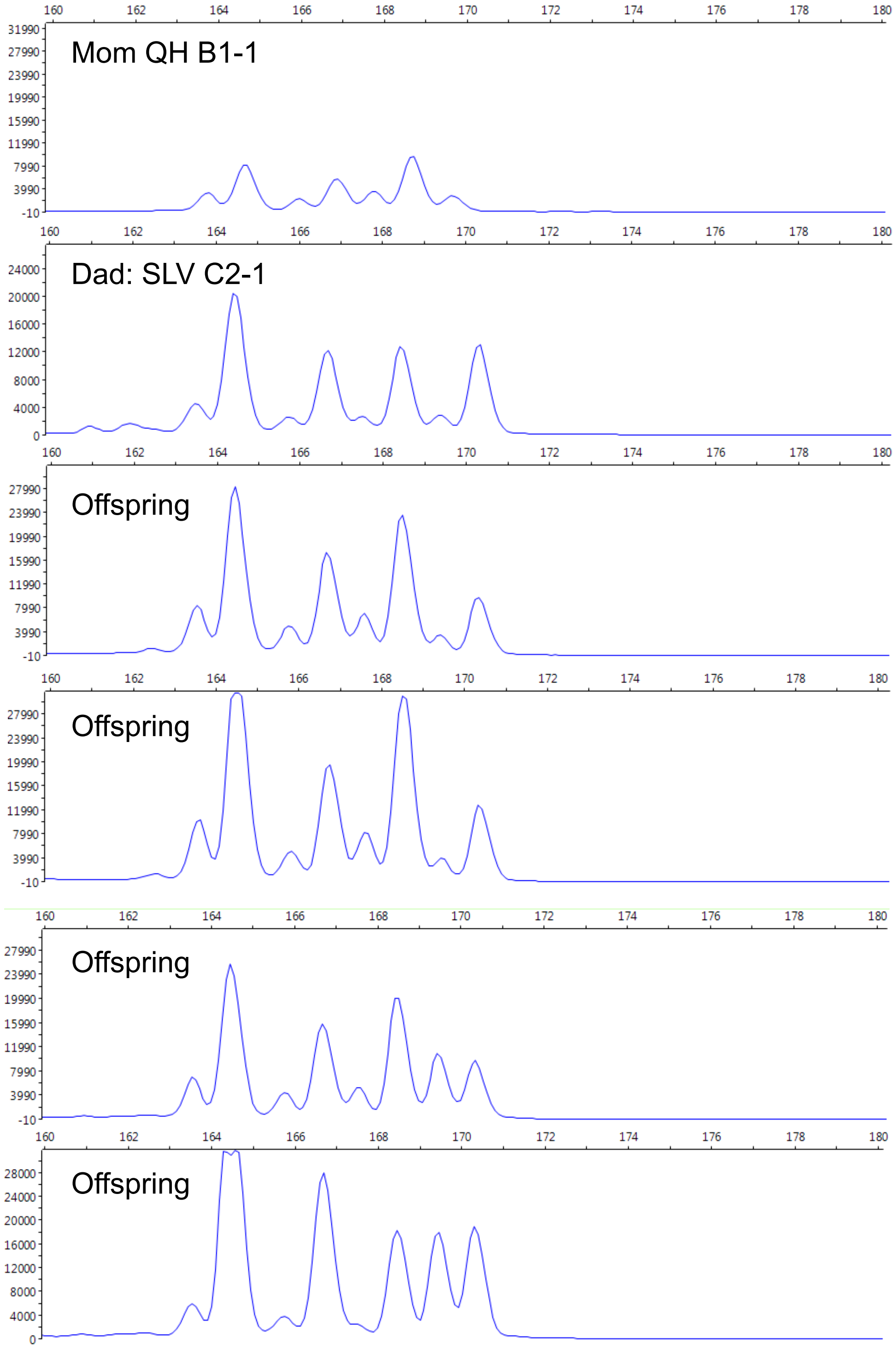

Mom QH B1-1
Dad: SLV C2-1
Offspring
Offspring
Offspring
Offspring

S1 Figure

M.

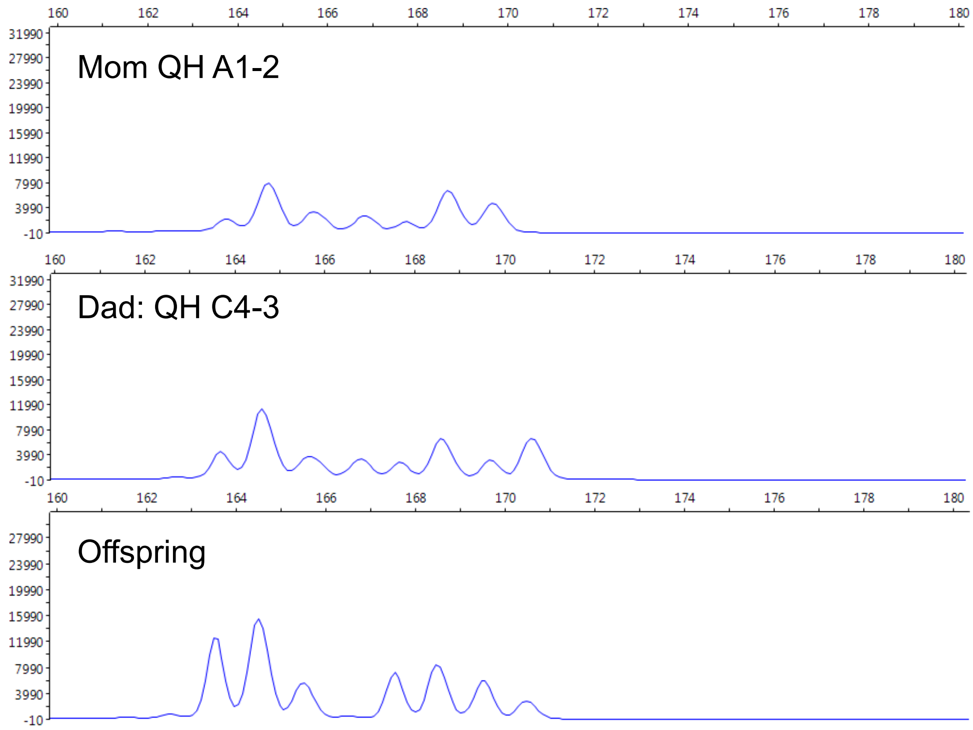

S1 Figure

N.

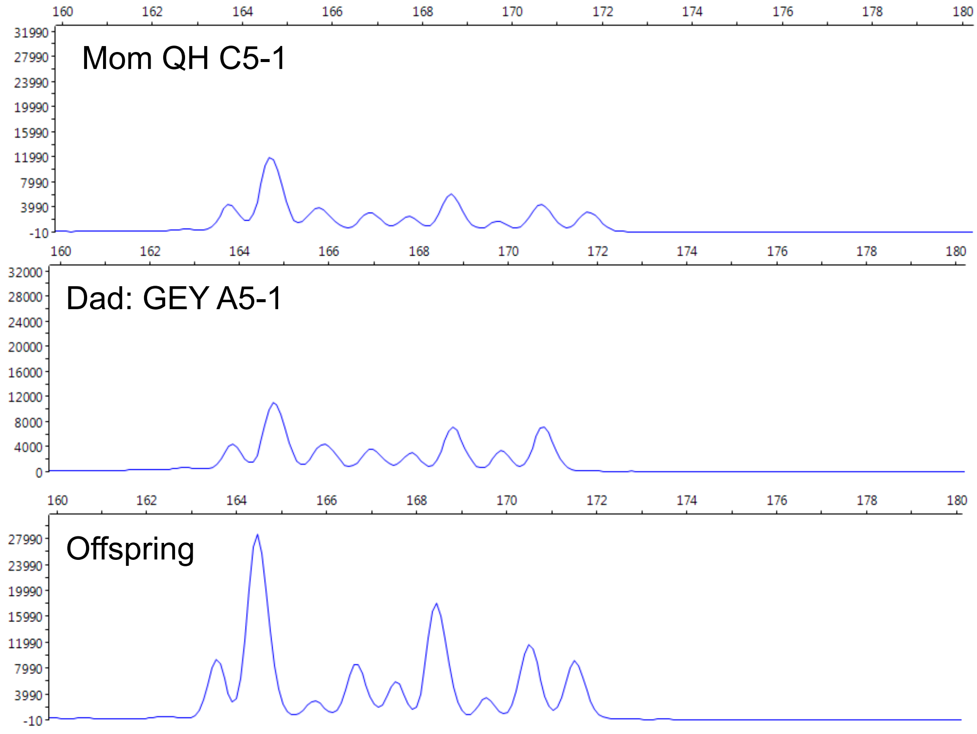

S1 Figure

O.

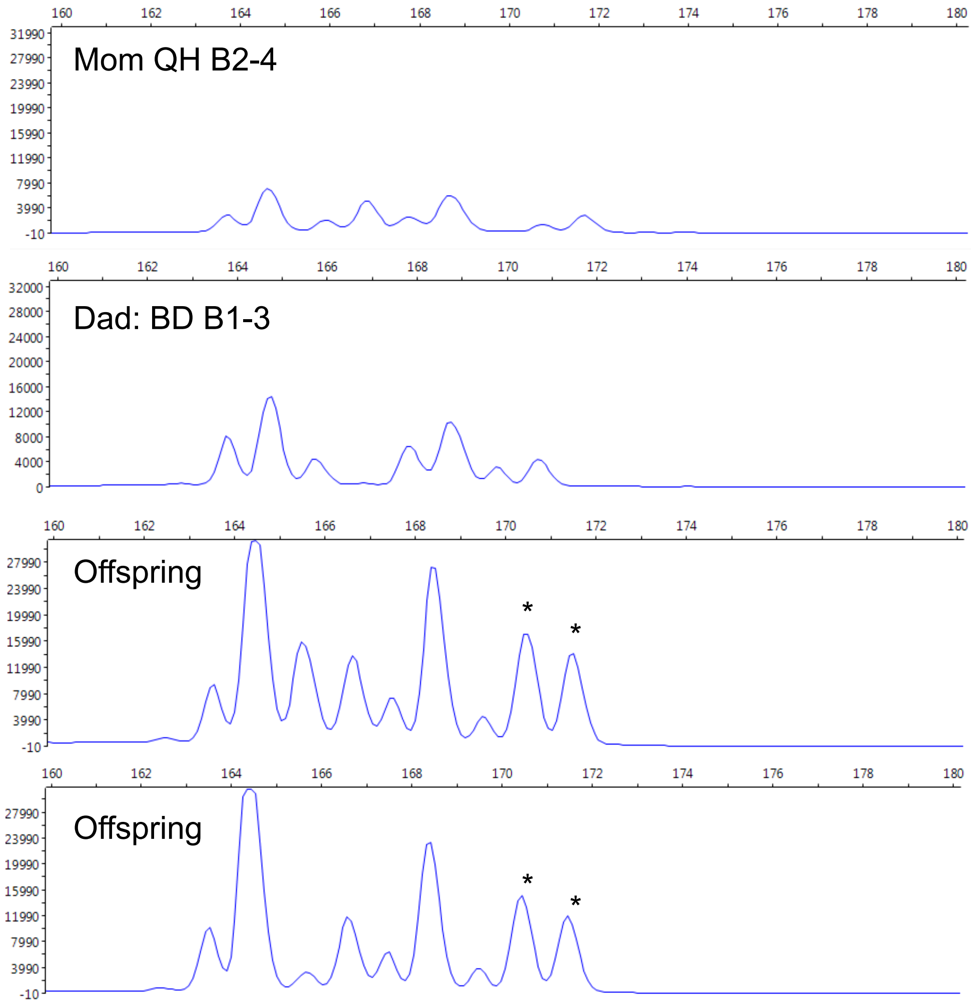

Supplement: S1 Fig — Parental and F1 chromatograms for locus D10 (A-E), locus D4/D4b (F-J), and locus C5 (K-O). The four fragments that appear in F1 individuals that are not present in the parents are indicated with asterisks. (PDF) [file pone.0227523.s001.pdf]
