# Supplementary figures and images for: Genome skimming and microsatellite analysis reveal contrasting patterns of genetic diversity in a rare sandhill endemic (Erysimum teretifolium, Brassicaceae)
Source: PLoS One. 2020 May 27;15(5):e0227523. doi: 10.1371/journal.pone.0227523 (PMC7252598; doi:10.1371/journal.pone.0227523)

S2 Figure

A.

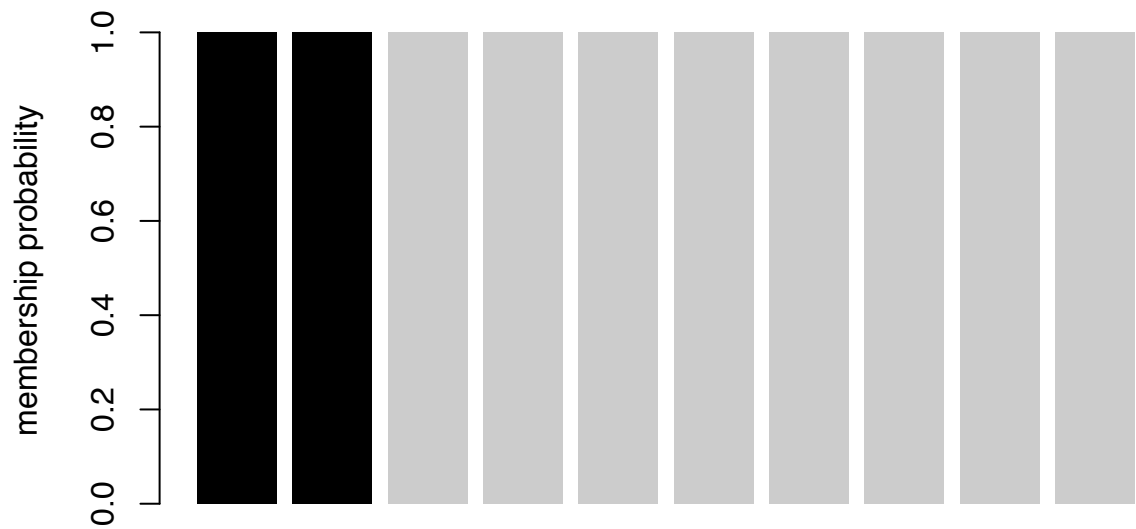

B.

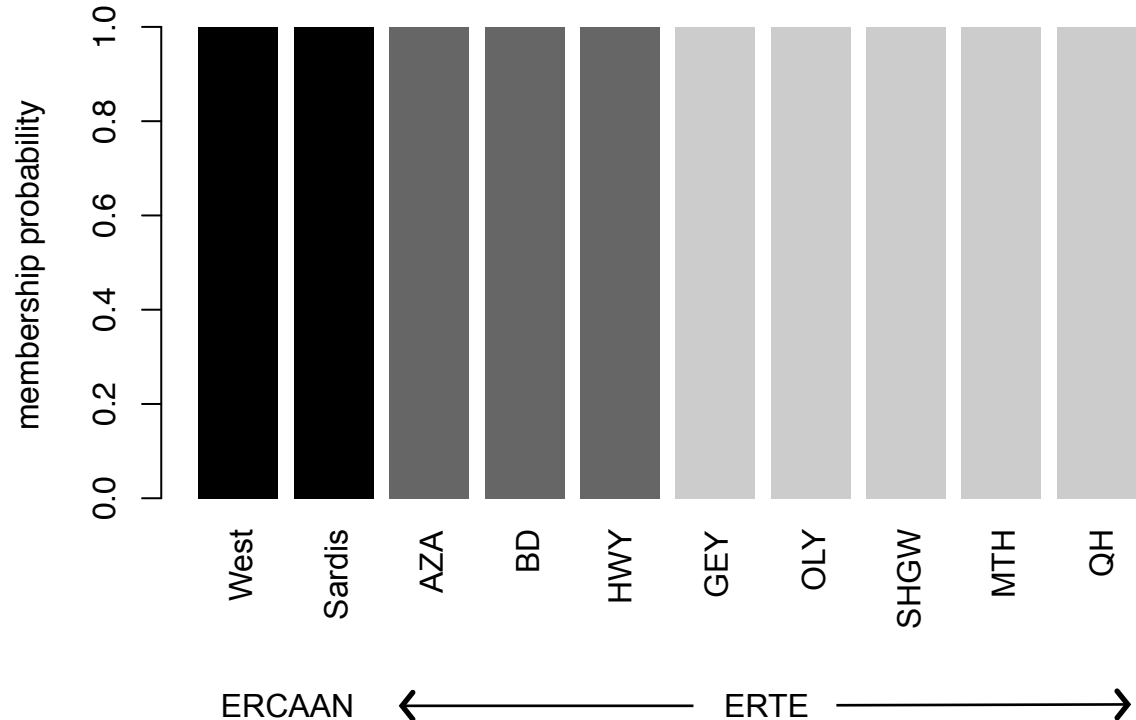

Supplement: S2 Fig — Two groupings (A) unambiguously differentiate ERCAAN (dark grey) and ERTE (light grey) populations. Three groupings (B) were based on the two distinct lineages recovered in the phylogenetic analyses of the chloroplast genomes (ERCAAN = dark grey; AZA, BD and HWY populations = grey; GEY, OLY, SHGW, MTH and QH populations = light grey). Population abbreviations are defined in Table 1. (PDF) [file pone.0227523.s002.pdf]

# S3 Figure

A.

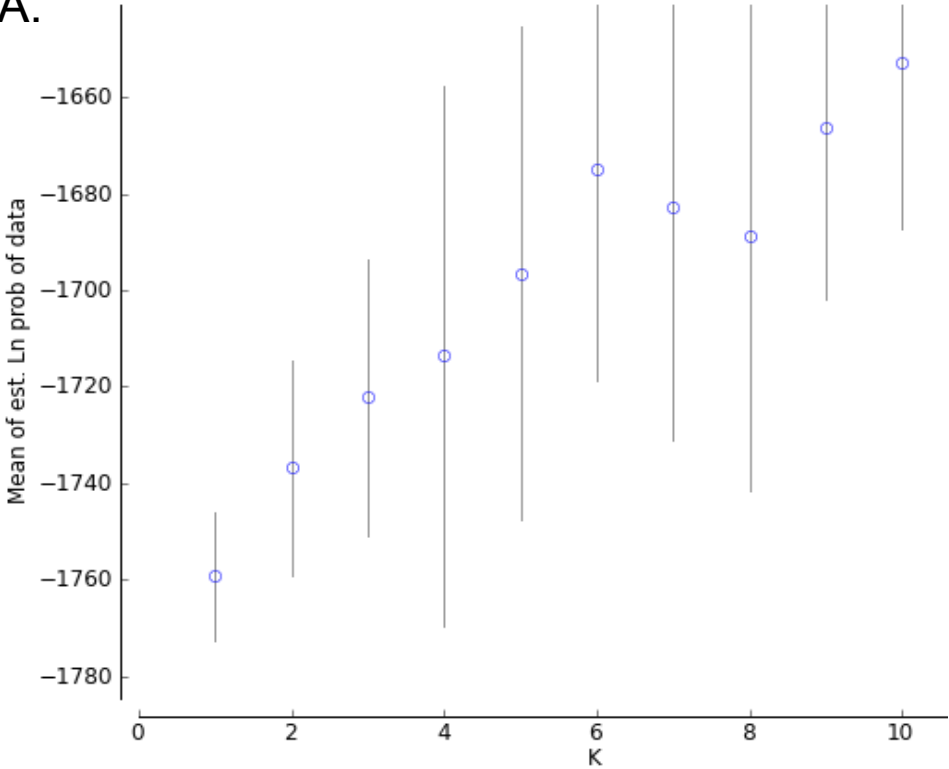

B.

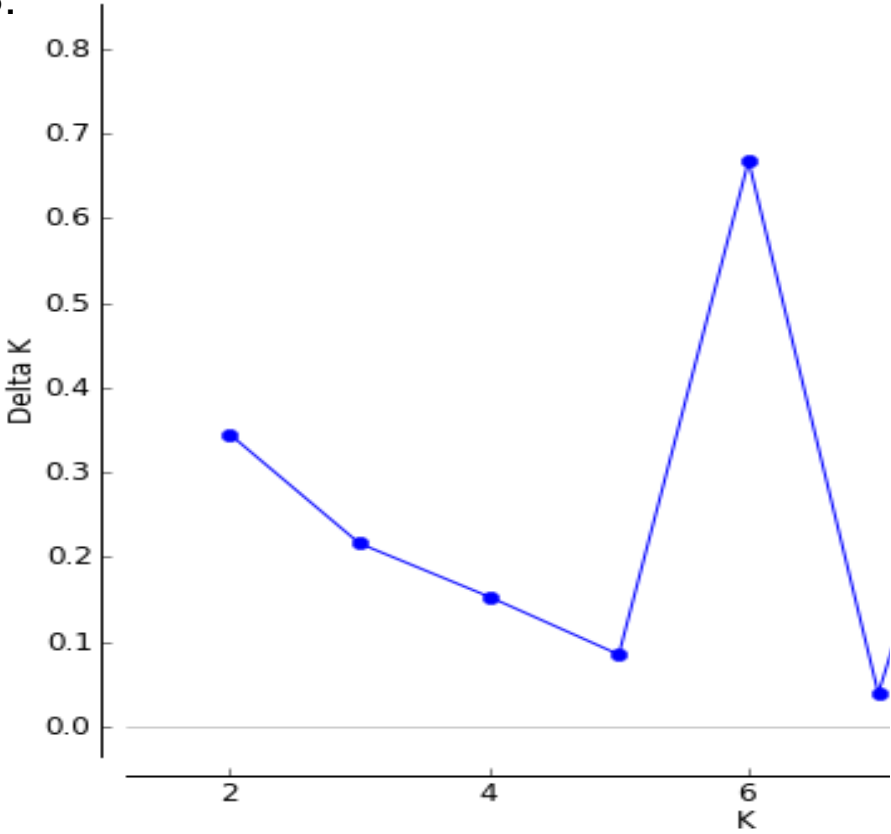

Supplement: S3 Fig — Using only samples from the eight populations of E. teretifolium, Structure Harvester identified k = 6 as the most likely grouping. Genetic groupings of k = 2 is also considered. Likelihood of K is indicated in A and ΔK is indicated in B. (PDF) [file pone.0227523.s003.pdf]
