# Supplementary material for: Genome skimming and microsatellite analysis reveal contrasting patterns of genetic diversity in a rare sandhill endemic (Erysimum teretifolium, Brassicaceae)
Source: PLoS One. 2020 May 27;15(5):e0227523. doi: 10.1371/journal.pone.0227523 (PMC7252598; doi:10.1371/journal.pone.0227523)
Supplement: S1 Table — (DOCX) [file pone.0227523.s004.docx]

**S1 Table. Microsatellite fragment diversity, sizes, and optimized annealing temperature.**

| Locus | Primer name ^a^ | Type of microsatellite repeat | Range of fragment sizes (bp) | Optimized annealing temperature (˚C) | Total number of fragments scored | Number of fragments used in analyses ^c^ |
| --- | --- | --- | --- | --- | --- | --- |
| C5 | C5 | Dinucleotide ^b^ | 164-168 | 56 | 5 | 3 |
| D4 | D4 | Trinucleotide | 192-216 | 57 | 9 | 8 |
| D4b | D4 | Trinucleotide | 330-340 | 57 | 1 | 1 |
| D10 | D10 | Trinucleotide | 180-210 | 60 | 9 | 8 |

^a^ Primer names are from Muñoz-Pajares et al. [43]

^b^ In *Erysimum mediohispanicum*, C5 is a trinucleotide repeat [43]. Here it is a dinucleotide repeat.

^c^ Four fragments were removed after comparing inheritance of fragments in parents and F1 offspring of controlled crosses
