# Supplementary material for: Genome skimming and microsatellite analysis reveal contrasting patterns of genetic diversity in a rare sandhill endemic (Erysimum teretifolium, Brassicaceae)
Source: PLoS One. 2020 May 27;15(5):e0227523. doi: 10.1371/journal.pone.0227523 (PMC7252598; doi:10.1371/journal.pone.0227523)
Supplement: Attachment — Submitted filename: rebuttal letter.PONE-D-19-33086.jbw.pdf [file pone.0227523.s005.pdf]

Santa Clara University

December 19, 2019

Dear PLOS ONE Editors,

Please consider our new manuscript entitled, “Genome skimming and microsatellite analysis reveal contrasting patterns of genetic diversity in a rare sandhill endemic (*Erysimum teretifolium*, Brassicaceae)”. It is a substantially improved study than the one we submitted in 2014 (PONE-D-14-47681). The newly submitted version includes:

- A new title
- A new first author
- A vast amount of new data from genome skimming
- Improved additional analyses for genome skimming and the microsats
- Completely revised Discussion including a section on “Genome sizing and microsats in polyploids”

As requested, this “rebuttal letter” addresses both of the previous reviewers’ comments in light of our new manuscript. Note: I have broken up Reviewer #1’s single large paragraph into sections to address each point individually. Our responses are in **bold**. If you have any further questions or concerns, please email me ASAP (jwhittall@scu.edu) or call my cell (831 332 3389).

Justen Whittall

December 21, 2014
PONE-D-14-47681
Mating system trumps island-like habitat in determining the distribution of genetic diversity in a rare sandhill endemic (Erysimum teretifolium, Brassicaceae)

Dear Dr. Whittall,

Thank you for submitting your manuscript to PLOS ONE. After careful consideration, we have decided that your manuscript does not meet our criteria for publication and must therefore be rejected.
…
Specifically, both reviewers are in agreement that by including only 4 co-dominant microsatellite markers in your analysis, there is insufficient strength of signal to draw any robust conclusions about genetic structure in your target populations.

The new manuscript adds the entire chloroplast genome, nuclear ribosomal cistron and an attempt at sequencing the mtDNA genome to the pre-existing microsatellite results. With this vast amount of additional data, the manuscript no longer suffers from “insufficient strength of signal to draw any robust conclusions…”. In fact, the opposite has appeared where we have strong signal from cpDNA that is in conflict with the nuclear DNA suggesting something interesting and new. Furthermore, as

Department of Biology
500 El Camino Real, Santa Clara, California 95053-0268
408-921-7035 FAX 408-554-2710

Santa Clara University

we describe above, since the microsatellites are treated as dominant markers (presence/absence), we have functionally 20 loci, not just four. Our inclusion of a positive control to test the power of these markers to differentiate a close relative UNDENIABLY shows that if there was deep genetic divergence within *E. teretifolium*, we would have detected it with these markers. Hopefully the combination of substantial amounts of new data from genome skimming and a clarification on the number and power of the nuclear markers comes across clearly and succinctly.

I am sorry that we cannot be more positive on this occasion, but hope that you appreciate the reasons for this decision.

Yours sincerely,

Owain Rhys Edwards, Ph.D.
Academic Editor
PLOS ONE

Reviewers' comments:

Reviewer #1: This study proposes to test whether mating system or population structure is more important in determining the genetic structure of a rare plant that has an island-like distribution based on habitat fidelity. The specific hypothesis tested that is that if population structure is important by limiting gene flow, genetic variation will be partitioned among populations, while if the SI breeding system is important there will be relatively little differentiation among populations. This is not novel investigation, with there being a very, very, large amount if literature available on this kind of topic and this study probably adds relatively little to what we know.
My understanding is that this is NOT a criterion for PLOS ONE. If I have misunderstood the underlying philosophy of the journal, please clarify.

One key issue to think about is what value of FST would have been enough to suggest to the authors that there was significant genetic structure, also how can they use their analysis framework to look for interactions between these two possible drivers of structure?
Fst, like most statistics can be tested for significance. We include a statistical test for significance of the Fst values (authors' lines 431-438). In this case, the test is whether the value is significantly different from zero (no structure) and sometimes testing for significant Fst values between individual populations.

Additional challenges are introduced by the choice of a hexaploid species (auto or allo we don't get to know) as a model system. This significantly complicates the analytical framework as making predictions of genotype distributions for multiple loci under

Department of Biology
500 El Camino Real, Santa Clara, California 95053-0268
408-921-7035 FAX 408-554-2710

random mating and looking for agreement with these using a co-dominant marker system that doesn't show dosage effects easily is incredibly difficult. Unfortunately the analytical framework chosen of treating the markers as presence-absence is not a powerful one with which to conduct the analyses attempted. In addition, the hexaploid genome combined with a dominant analytical model significantly reduces the power to assess population differentiation by biasing genetic variation values for population up, as demonstrated by several comparative analyses of genetic diversity and structure in several other herbaceous species.
We cannot change the ploidy of this species, nor can we change the fact that it is critically endangered. What we can change is the number and type of markers, which we have using genome skimming. The subsequent analytical methods are much more powerful for SNP data from the cpDNA and nuclear ribosomal cistron (e.g., Maximum Likelihood Phylogenetic Analysis in Figure 2 and Discriminant Analysis of Principle Components in Figure 3).

As described in this new manuscript, this endangered species' future rests (in part) on our estimate of the distribution of genetic diversity. If genetic diversity is mostly within populations, we should preserve a few large populations. If genetic diversity is mostly among populations, we should preserve more populations, especially those that are genetically unique. Whether we have contributed to this question in a scientifically rigorous way is a question I will leave to your reviewers to determine. I argue that we have discovered that the cpDNA has a different evolutionary history than the nuclear genome and both should be considered before making any final management decisions. If there was deep divergence in the nuclear genome, our 20 nuclear loci (microsats treated as present/absent) in combination with the nuclear ribosomal cistron sequences would have detected it.

Finally the interpretation of the final results in a conversation context are not strong.
Assuming the reviewer is commenting on the Discussion, it is largely rewritten to interpret the results of the cpDNA data with the nuclear data. We have attempted to formalize the style to avoid anything that might be interpreted as "conversation context".

Reviewer #2: The manuscript, PONE-D-14-47681, is an investigation of how breeding system has impacted the distribution of genetic diversity in a rare, edaphically specialized mustard. The manuscript is well constructed, well written, and easy to follow. I appreciate the analyses that were conducted to examine inheritance of microsatellite loci, and the analyses of genome size. These types of analyses should be conducted in more studies where polyploidy is assumed to be occurring and the authors have provided a good model for how to carry out these types of analysis.

The major limitation of this research is that only 4 microsatellite loci were included in the study. In reviewing the analyses and results, I am concerned that you do not have enough signal in your data to support the conclusions that have been reached. This is particularly evident when examining the Structure Harvester results (Table S2, Figure S3) and

Department of Biology
500 El Camino Real, Santa Clara, California 95053-0268
408-921-7035 FAX 408-554-2710

Structure results (Figure 2). Related to the Structure Harvester results, the ΔK values are all below 1. Although there is some modest differences among K groups, the theoretical values of ΔK from the Evanno paper, on which Structure Harvester is based, are all greater than 30 and I have not seen any published values of ΔK less than 10. In examining these results it seems that there is no improvement in increasing the number of K groups. The lack of signal is also evident in the Structure results for K=6 and K=8. The fact that groups are all divided laterally within populations is frequently an indication of a lack of signal, where the algorithm divides individual genotypes relatively evenly among clusters.
We have added over 150,000 additional base pairs from multiple genomes to the microsatellite data. Furthermore, for clarification, when we treat the four microstatellites as dominant data, there are actually 20 loci and each is treated as presence/absence (Results, lines 385-395). We too were concerned about the possibility of low power to detect population structure. Therefore, we included a "positive control" comparing our 20 "loci" to a very closely related species that is morphologically and geographically distinct, *E. capitatum var. angustatum* (lines 398-407). Briefly, we found that the four microstatellites treated as dominant markers were able to clearly differentiate our target taxon, *E. teretifolium*, from the very closely related *Erysimum capitatum* var. *angustatum* (Fig. 2A). If there was deep genetic divergence in the nuclear genome within *E. teretifolium*, we had the power to detect it and we didn't. This is an important result in light of the cpDNA data that identified a deep split in the plastome's evolutionary history. Thus, we have chosen to keep the microsatellites to supplement the genome skimming results in the new manuscript. Finally, to directly address the reviewer's concerns, we have added a section to the Discussion on the challenges of using microsatellites in hexaploids ("Genome sizing and microsatellites in polyploids", lines 634—656). In any case, all analyses pointed to the presence of a core cluster of populations that are composed largely of admixed individuals with low probability of assignment to any single genetic grouping.

In my opinion, the Structure results are not reliable, which also impacts the AMOVA analyses, which have utilized the structure groupings. These two analyses, Structure and AMOVA, are the foundation for the manuscript in its current form. Unfortunately, the lack of signal in the structure results also leads me to question the accuracy of the pairwise Fst values.
Although we stand behind our STRUCTURE analysis (see response immediately above), our AMOVA analysis focuses on the distribution of genetic diversity within and among populations. Since >85% of variation exists within populations, how we split out the remaining <15% is relatively inconsequential. Therefore we don't feel the AMOVA results would be compromised if the STRUCTURE analysis suffered from low power. If the editor or reviewers prefer, we can modify the AMOVA to just within vs. among populations.

I am also concerned about including multiple samples from the four populations where maternal families where the sampling unit. My concern is that we cannot assume that the sampled offspring would have survived in the wild, which will impact the partitioning of

Department of Biology
500 El Camino Real, Santa Clara, California 95053-0268
408-921-7035 FAX 408-554-2710

Santa Clara University

diversity in AMOVA analyses. I would recommend rerunning the analyses using only 1 individual to represent each maternal family.

I agree that sampling individuals from seed is suboptimal, but when faced with an endangered species on the brink of extirpation at multiple sites, we did what we could to include them (vs. leaving them out because we did not have sufficient leaf samples or sampling leaves was not allowed by agency folks). If this sampling affected the results, we'd expect to see different levels of genetic diversity in these populations than in the populations sampled from wild grown individuals. This is not the case (see genetic diversity results reported in Tables 1 and Table 3 and in the associated Results section entitled, "Partitioning of genetic diversity and isolation by distance", lines 421, 438). Furthermore, the number of loci under selection in the wild is surely a relatively small target compared to the size of the *E. teretifolium* genome (2.86 billion base pairs, lines 311-312) and VERY UNLIKELY to be linked to the 20 microsatellite "loci" we report herein.

A final recommendation is to include a table of the AMOVA results. Although your pie chart is visually nice, it is best to be able to view the variance and F-statistics associated with each level of the comparison.

Done (see Table 2).

Thank you for your consideration,

Justen Whittall
jwhittall@scu.edu
(831) 332 3389

Department of Biology
500 El Camino Real, Santa Clara, California 95053-0268
408-921-7035 FAX 408-554-2710
