# Supplementary material for: Genome skimming and microsatellite analysis reveal contrasting patterns of genetic diversity in a rare sandhill endemic (Erysimum teretifolium, Brassicaceae)
Source: PLoS One. 2020 May 27;15(5):e0227523. doi: 10.1371/journal.pone.0227523 (PMC7252598; doi:10.1371/journal.pone.0227523)
Supplement: Attachment — Submitted filename: rebuttal letter PONE-D-19-33086.2nd revision.200417.docx [file pone.0227523.s007.docx]

PONE-D-19-33086R1
Rebuttal Letter Accompanying 2nd Revision of:

“Genome skimming and microsatellite analysis reveal contrasting patterns of genetic diversity in a rare sandhill endemic (*Erysimum teretifolium*, Brassicaceae)”

Review Comments to the Author

Please use the space provided to explain your answers to the questions above. You may also include additional comments for the author, including concerns about dual publication, research ethics, or publication ethics. (Please upload your review as an attachment if it exceeds 20,000 characters)

Reviewer #1: I have revised the detailed cover letter and the new version of the manuscript, and the authors have addressed all the points raised in my review, providing a reasonable reply when my suggestions have not been followed. I particularly acknowledge the document with the track-changes activated, which greatly facilitates the revision. I fully support publication of the manuscript in its present form.

Reviewer #3: Del Valle et al. studied 8 island-like populations of Erysimum teretifolium and found significant effect of isolation by distance. The phylogenies were reconstructed by cpDNA and nrDNA and revealed two groups with high bootstrap support. Comprehensive microsatellite analyses were conducted, two of the populations were suggested for conservation. The manuscript is a revised version. It is well-written and most of the patterns are discussed in detail. However, there were still a few points need to be clarified. I listed these points as follows.

1. The phylogenies of cpDNA and nrDNA indicated different grouping of these populations, possibly caused by inconsistent maternal and paternal histories. Also, both trees did not support affinity between QH and BD. Need more clarification for these.

**Although Figure 2A (cpDNA) and 2B (nrDNA) show different topologies as noted by the reviewer, the nrDNA phylogenetic analysis shows no branches within the ERTE clade with bootstrap support >50%. Thus, there is no incongruence between the cpDNA and nrDNA phylogenies since the nrDNA tree only supports the monophyly of ERTE to the exclusion of the ERCAAN samples. A discussion about maternal and paternal evolutionary histories would suggest the nrDNA topology is strongly supported. We tried to improve this message in the Genome Skimming portion of the Results entitled, “*Analysis of the nrDNA alignment*” where we state: “Maximum likelihood phylogenetic analysis using RAxML produced a tree with only a single well-supported branch defining the monophyly of the ERTE samples as separate from the two ERCAAN samples (BS = 100%; Fig 2B).” To help clarify this previously stated result, we have added a clarifying sentence to the last paragraph in the Discussion section on “Genome skimming…” that now states, “Although the cpDNA tree topology appears incongruent with that of the nrDNA region, the latter lacks any branches within ERTE with bootstrap values > 50%.”**

**Thank you for pointing out the inconsistency between the cpDNA tree (Fig. 2A) and the microsatellite affinities (*e.g.,* Fig. 4B), especially with regard to QH and BD where seeds may have been moved unofficially. We now raise this point in the Discussion section entitled “Island-like patterns…” where we previously discussed the possibility of “unofficial trafficking” of seeds between QH and BD in regards to the microsatellite data alone. The statement is now followed by this phrase, “however BD and QH fall into separate, strongly supported cpDNA lineages (Fig. 2A).” We have added a comparable qualifying statement in the second paragraph of the Discussion section entitled, “Conservation implications”.**

2. Structure result at K=6 indicated HWY and SHGW possessing most distinct genetic components, and the authors suggested these two populations are important targets for conservation. Generally I agree with this point. By the way, the result at K=2 indicated SHGW and QH possessing distinct components while the other populations being admixtures of them. According to the location of populations and the dispersal direction suggested by wind (as mentioned from northwestern), it seems to me that the QH is an important population in this sense. Moreover, the information that BD might be a clonal population by QH also support this. At the result of K=6, although the identity of QH is blurred by multiple genetic components, I still suggested this population could be further discussed.

**We agree that in the K=2 Structure analysis of the microsatellite data, the most genetically divergent populations are SHGW and QH (and BD looks very similar to QH). Thank you for pointing out the need to include QH as a priority for preserving the remaining genetic variation based on the microsatellite results.**

**We have emphasized the evidence for the genetic similarity of QH and BD revealed by the K=2 Structure analysis in the Discussion section of “Island-like patterns…” in the 1st full paragraph. It now reads (underlined = new), “Structure analysis of the microsatellite data (K = 2) suggests BD and QH are very similar genetically – most individuals from both populations have the same proportions of admixture (Fig 4B).” Furthermore, we have added QH to the list of populations with unique genetic variation. The sentence in the “Conservation implications” section of the Discussion now read, “Yet, in order to capture the remaining genetic variation, peripheral populations (such as QH, HWY, and SHGW) must also be preserved.”**

3. The map of population distribution (Fig 1B) needs an indicator for north.

**Done.**
